# *CPSF1* mutations are associated with early-onset high myopia and involved in retinal ganglion cell axon projection

**DOI:** 10.1093/hmg/ddz029

**Published:** 2019-01-26

**Authors:** Jiamin Ouyang, Wenmin Sun, Xueshan Xiao, Shiqiang Li, Xiaoyun Jia, Lin Zhou, Panfeng Wang, Qingjiong Zhang

**Affiliations:** State Key Laboratory of Ophthalmology, Zhongshan Ophthalmic Center, Sun Yat-sen University, 54 Xianlie Road, Guangzhou 510060, China

## Abstract

High myopia is a severe form of nearsightedness, which can result in blindness due to its associated complications. While both genetic and environmental factors can cause high myopia, early-onset high myopia (eoHM), which is defined as high myopia that occurs before school age, is considered to be caused mainly by genetic variations, with minimal environmental involvement. Here we report six rare heterozygous loss-of-function (LoF) variants in *CPSF1* that were identified in six of 623 probands with eoHM but none of 2657 probands with other forms of genetic eye diseases; this difference was statistically significant (*P* = 4.60 × 10^−5^, Fisher’s exact test). The six variants, which were confirmed by Sanger sequencing, were c.3862_3871dup (p.F1291^*^), c.2823_2824del (p.V943Lfs^*^65), c.1858C>T (p.Q620^*^), c.15C>G (p.Y5^*^), c.3823G>T (p.D1275Y) and c.4146-2A>G. Five of these six variants were absent in existing databases, including gnomAD, 1000G and EVS. The remaining variant, c.4146-2A>G, was present in gnomAD with a frequency of 1/229918. Clinical data demonstrated eoHM in the six probands with these mutations. Knockdown of *cpsf1* by morpholino oligonucleotide (MO) injection in zebrafish eggs resulted in small eye size in 84.38% of the injected larvae, and this phenotype was rescued in 61.39% of the zebrafish eggs when the *cpsf1* MO and the *cpsf1* mRNA were co-injected. The projection of retinal ganglion cell (RGC) towards the tectum was abnormal in *cpsf1* morphants. Thus, we demonstrated that heterozygous LoF mutations in *CPSF1* are associated with eoHM and that *CPSF1* may play an important role in the development of RGC axon projection.

## Introduction

Myopia, also known as nearsightedness, is a public health problem worldwide, especially in Asia, as it affects 80–90% of teenagers and young adults in urban areas of East and Southeast Asia (1–3), and the prevalence of myopia has increased in recent years (4,5). High myopia is a severe form of myopia characterized by a spherical refraction measurement of at least −6.00 D or an axial length of 26 mm. High myopia is one of the leading causes of blindness due to its complications, such as retinal detachment, choroidal neovascularization, posterior staphyloma, glaucoma, cataracts and retinopathy (6–8). Genetic factors have been demonstrated to contribute to the development of high myopia (9–16). High myopia can be classified into early-onset and late-onset forms: (1) early-onset high myopia (eoHM) presents before school age and has minimal environmental effects, so genetic factors are considered to play major roles; and (2) late-onset high myopia (loHM) develops gradually from mild to high myopia, usually starting in primary school, and is closely associated with extensive near work. Genetic studies on myopia have been popular in recent years (11). Genome-wide association studies on high myopia, including both eoHM and loHM, have identified a series of relevant single-nucleotide polymorphisms (SNPs) (17,18), but the exact molecular mechanisms associated with these SNPs are mostly unknown.

Genetic factors are considered to be a determining factor in the development of eoHM (19–22). Therefore, eoHM should be a unique resource in searching for genes responsible for myopia. To date, mutations in ten genes have been identified by linkage analysis combined with whole-exome sequencing (WES) or WES alone, including five genes related to autosomal dominant eoHM [*ZNF644* (OMIM 614159) (23), *SCO2* (OMIM 604272) (24), *P4HA2* (OMIM 600608) (25), *SLC39A5* (OMIM 608730) (26) and *BSG* (OMIM 109480) (27)], three genes related to autosomal recessive eoHM [*LRPAP1* (OMIM 104225) (28), *LEPREL* (OMIM 610341) (29) and *LOXL3* (OMIM 607163) (30)] and two genes related to X-linked eoHM [*ARR3* (OMIM 301770) (31) and *OPN1LW* (OMIM 300822) (32)]. However, mutations in these genes could be identified in less than 10% of patients with eoHM (27,30–33). Additionally, mutations in genes listed in RetNet (https://sph.uth.edu/retnet/) could be detected in approximately one-fourth of families based on our previous studies (34,35). Therefore, the genetic factors underlying about 65–70% cases of eoHM have not yet been identified.

In the current study, comparative analysis of our in-house WES data revealed a novel candidate gene, *CPSF1*, for which loss-of-function (LoF) mutations were detected only in six probands with eoHM but not in those with other forms of genetic eye diseases.

*CPSF1* (OMIM 606027) encodes a subunit of the cleavage and polyadenylation specificity factor that plays an important role in processing the 3′ ends of eukaryotic mRNA precursors, resulting in the addition of the poly (A) tail (36). Recently, several studies performed in cell lines have reported that *CPSF1* may be related to lung cancer, ovarian cancer and prostate cancer (37–39), and *CPSF1* was also shown to affect definitive haematopoietic stem cell survival in a screen of ENU-mutated zebrafish (40). In addition, reduced *Cpsf1* expression was found in mice with a *Pax6* defect, which suggested that *Cpsf1* might be downstream of *Pax6* in mice (41). However, the relationship between *CPSF1* and human ocular diseases, including myopia, remains unknown. Following the identification of LoF mutations of *CPSF1* exclusively in eoHM, knockdown of *cpsf1* in zebrafish was performed in the current study, and the results suggested that *cpsf1* was involved in eye development and the axon-to-brain projection of retinal ganglion cells (RGCs). Together, these results suggest that LoF mutations in *CPSF1* may be a novel cause of eoHM, and highlight a potentially new mechanism for the disease.

## Results

### *CPSF1* mutations in eoHM

**Figure 1:**
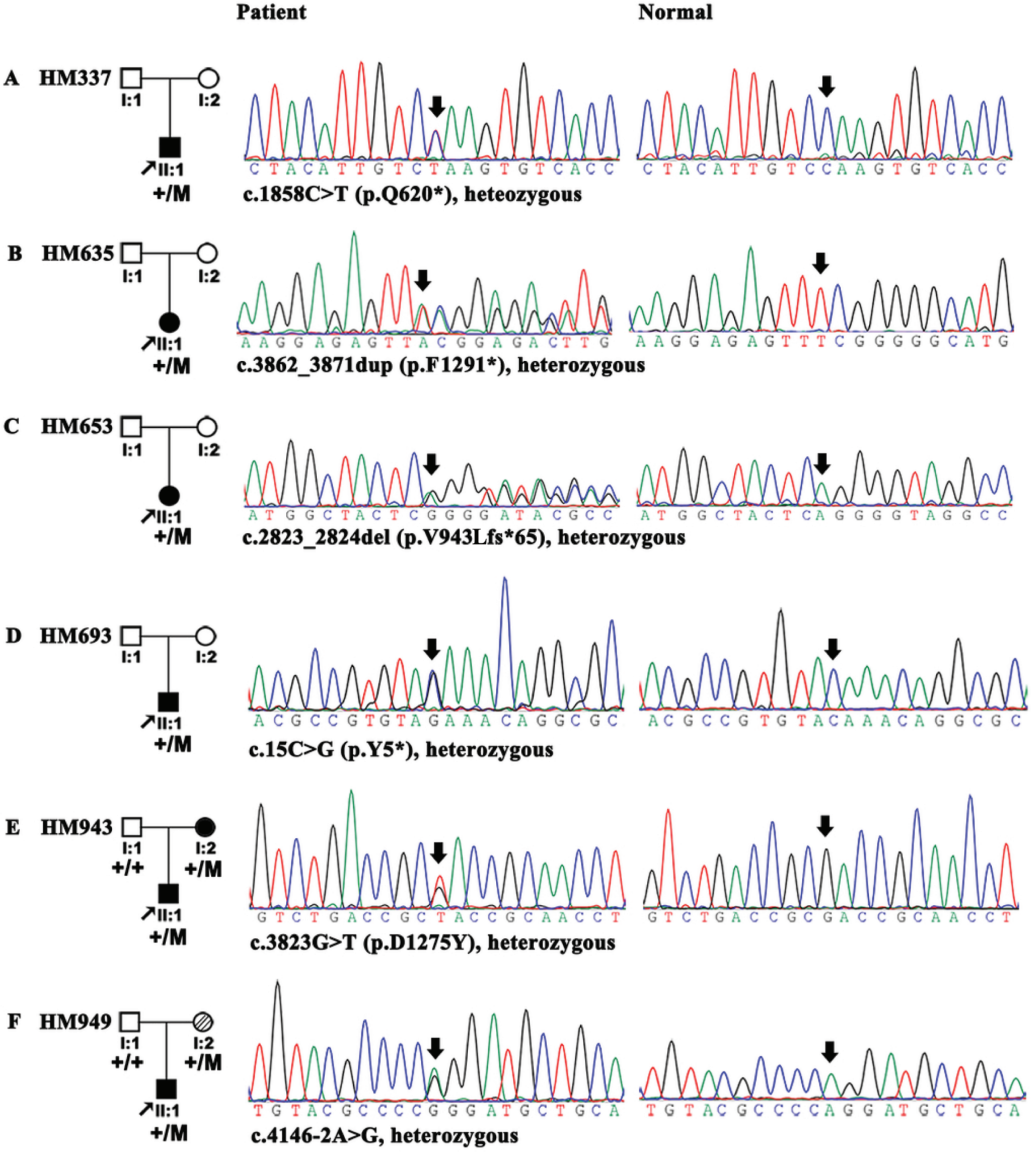
Mutations in *CPSF1* identified in six unrelated probands with eoHM. Pedigrees are shown in the left column. Sequences from probands with mutations were shown in the middle column, and sequences from normal controls were shown in the right column. For families HM943 and HM949, the mutations were identified from the proband and his mother. All of the mutations in the six probands were described under each sequence according to the nomenclature recommended by the Human Genome Variation Society (HGVS).

**Figure 2:**
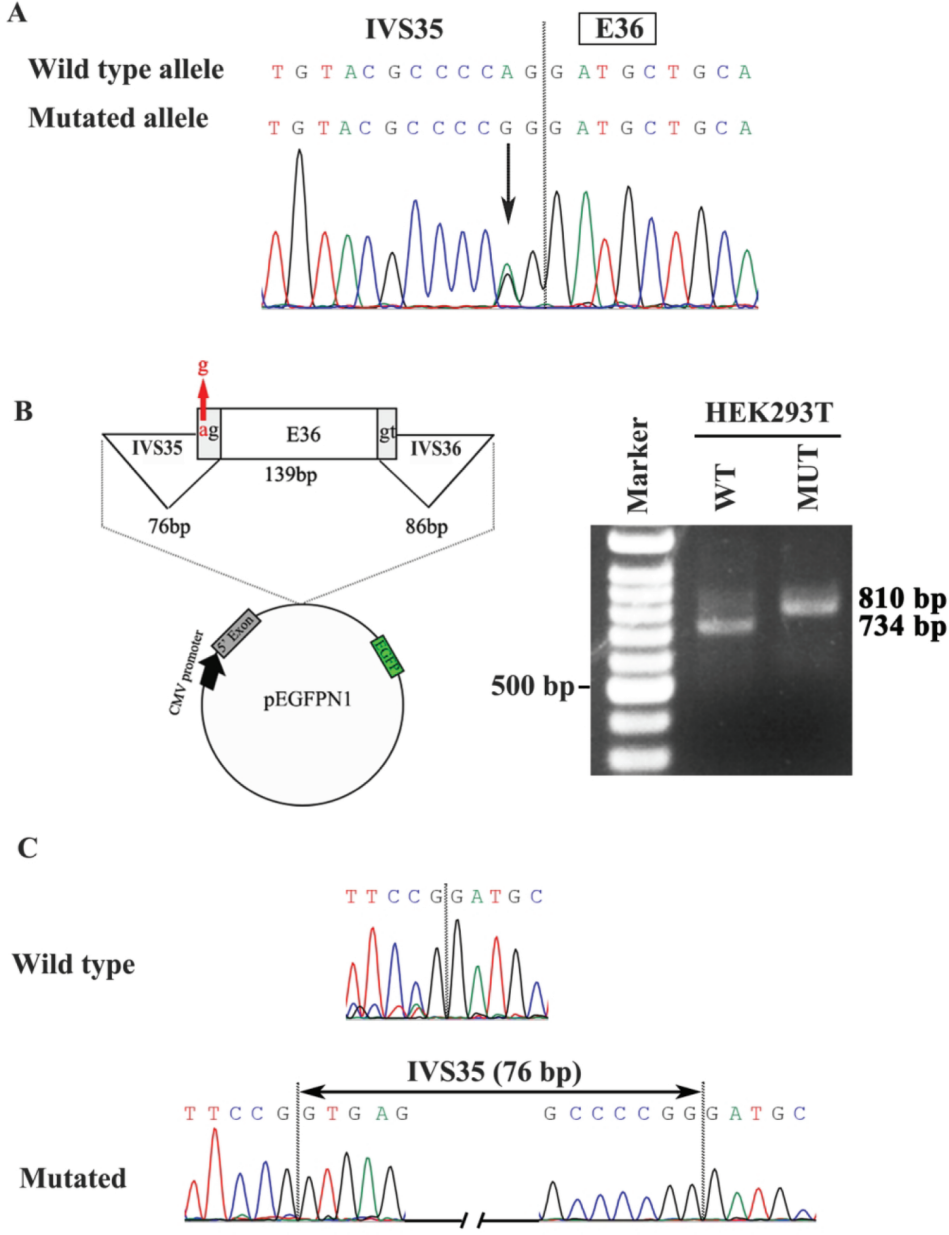
Minigene analysis of the effect of the c.4146-2A>G mutation for splicing. **(A)** The c.4146-2A>G mutation located at the boundary between intron 35 (IVS35) and exon 36 (E36). **(B)** Left, diagram of the minigene construction including exon 36 and flanking IVS35 and IVS36 by minigene analysis as well as pEGFPN1 construct. Red arrow, the location of the c.4146-2A>G mutation. Right, results of RT-PCR after transfection of pEGFPN1-WT vector containing the WT A allele and pEGFPN1-MUT containing the mutant G allele in HEK293T, respectively. **(C)** Sequences of cDNA by minigene analysis. The mutated allele results in the inclusion of IVS35 between E35 and E36 (the 76 bp sequence of whole IVS35 were shown in Supplementary Figure S6) while the WT allele does not include IVS35.

In a comparative analysis of WES data, six LoF variants in *CPSF1* were detected in six of 623 probands with eoHM, but none were detected in the 2657 probands with other forms of genetic eye diseases included 1139 with inherited retinal dystrophy, 812 with glaucoma and 706 with other genetic eye diseases; this difference in variant detection was significant (*P* = 4.60 × 10^-5^, Fisher’s exact test). All six variants were rare (Table 1, Fig. 1); five variants were novel and not present in existing databases (EVS, gnomAD, and 1000G), while the c.4146-2A>G mutation was present in gnomAD with a frequency of 1/229918 alleles. All variants are expected to cause LoF of the encoded *CPSF1*, including three nonsense variants (NM_013291.3; c.15C>G/p.Y5^*^, c.1858C>T/p.Q620^*^, and c.3862_3871dup/p.F1291^*^), one frameshift variant (NM_013291.3; c.2823_2824del/p.V943Lfs^*^65), a splicing-acceptor variant (NM_013291.3; c.4146-2A>G) and a missense variant (NM_013291.3; c.3823G>T/p.D1275Y) that was also predicted to affect splicing by ANNOVAR (http://www.fruitfly.org/seqtools/splice.html). The three nonsense variants and the one frameshift variant induce premature termination codons (PTCs), which are expected to result in nonsense-mediated decay (NMD) of the aberrant RNA transcript, leading to the absence of a protein product (null allele). NMD is usually avoided only when the PTC is located within the last exon or the last 50 bp of the second to last exon, and the four PTC variants were located in earlier exons (p.F1291^*^ in exon 34, p.V943Lfs^*^65 in exon 24, p.Q620^*^ in exon 18 and p.Y5^*^ in exon 1 of the 37-exon gene); thus, these variants were likely to result in the absence of a protein product. The other two mutations were predicted to result in aberrant splicing, including an intronic mutation (c.4146-2A>G) in IVS35 and an exonic mutation (c.3823G>T/p.D1275Y) in exon 33. Minigene analyses were performed to validate the effects of the two mutations on splicing. It was confirmed that the c.4146-2A>G allele (MUT) resulted in intron retention compared to the wild-type (WT) allele (Fig. 2). However, the mutant allele (T) and the WT allele (G) at c.3823 of exon 33 both caused retention of intron 32 (Supplementary Material, Fig. S2). The aspartic acid at 1275 was highly conserved among nine vertebrates (Supplementary Material, Fig. S2).

**Table 1:** Potential pathogenic variants detected in *CPSF1* (NM_013291.3)

**ID**	**Chr**	**Position**	**Nucleotide change**	**Effect**	**Status**	**PPH2/SS**	**SIFT**	**PROVEAN**	**1000G**	**EVS**	**GnomAD**	**Control**	**Known**
HM337	Chr8	145623728	c.1858C>T	p.Q620^*^	Het	NA	NA	NA	None	None	None	0/5314	Novel
HM635	Chr8	145619251	c.3862_3871dup	p.F1291^*^	Het	NA	NA	NA	None	None	None	0/5314	Novel
HM653	Chr8	145621815	c.2823_2824del	p.V943Lfs^*^65	Het	NA	NA	NA	None	None	None	0/5314	Novel
HM693	Chr8	145634528	c.15C>G	p.Y5^*^	Het	NA	NA	NA	None	None	None	0/5314	Novel
HM943	Chr8	145619364	c.3823G>T	p.D1275Y and splicing	Het	PrD	D	D	None	None	None	0/5314	Novel
HM949	Chr8	145618807	c.4146-2A>G	Splicing acceptor	Het	SSA	NA	NA	None	None	1/229918	0/5314	Known

Abbreviations: Het, heterozygous; del, deletion; dup, duplication; PrD, probably damaging; SSA, splicing acceptor; D, damaging; 1000G, 1000 Genomes; EVS, Exome Variant Server; GnomAD, genome aggregation database; NA, not applicable.

The six mutations were confirmed by Sanger sequencing (Fig. 1). Data from a segregation analysis of family members were obtained for families HM943 and HM949, but DNA samples were unavailable for the parents of the other four probands.

**Figure 3:**
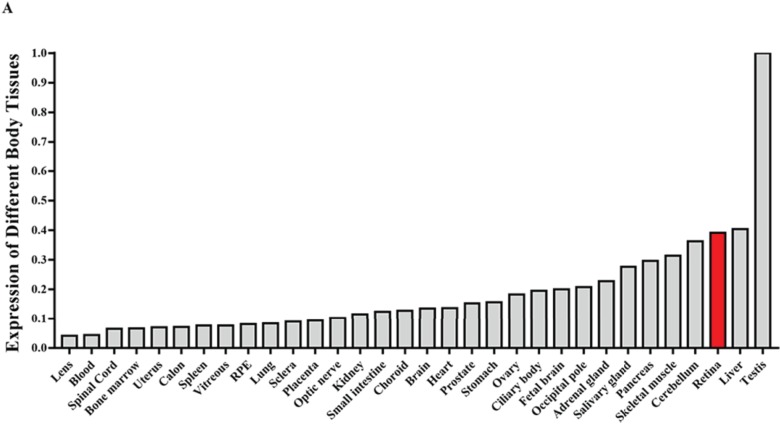
Expression analysis of *CPSF1* mRNA in human tissues. All of the RNA samples used in this study, except for the ocular samples, were acquired from at least three people, and they were extracted from 12 male and female Caucasian individuals who experienced sudden death at the age of 18 to 54 years. The ocular RNA samples used in this study, except for the retinal pigment epithelium (RPE) sample, were acquired from an eye donor who died of meningioma at 17 years old. The RPE sample was acquired from the ARPE-19 cell line.

**Figure 4:**
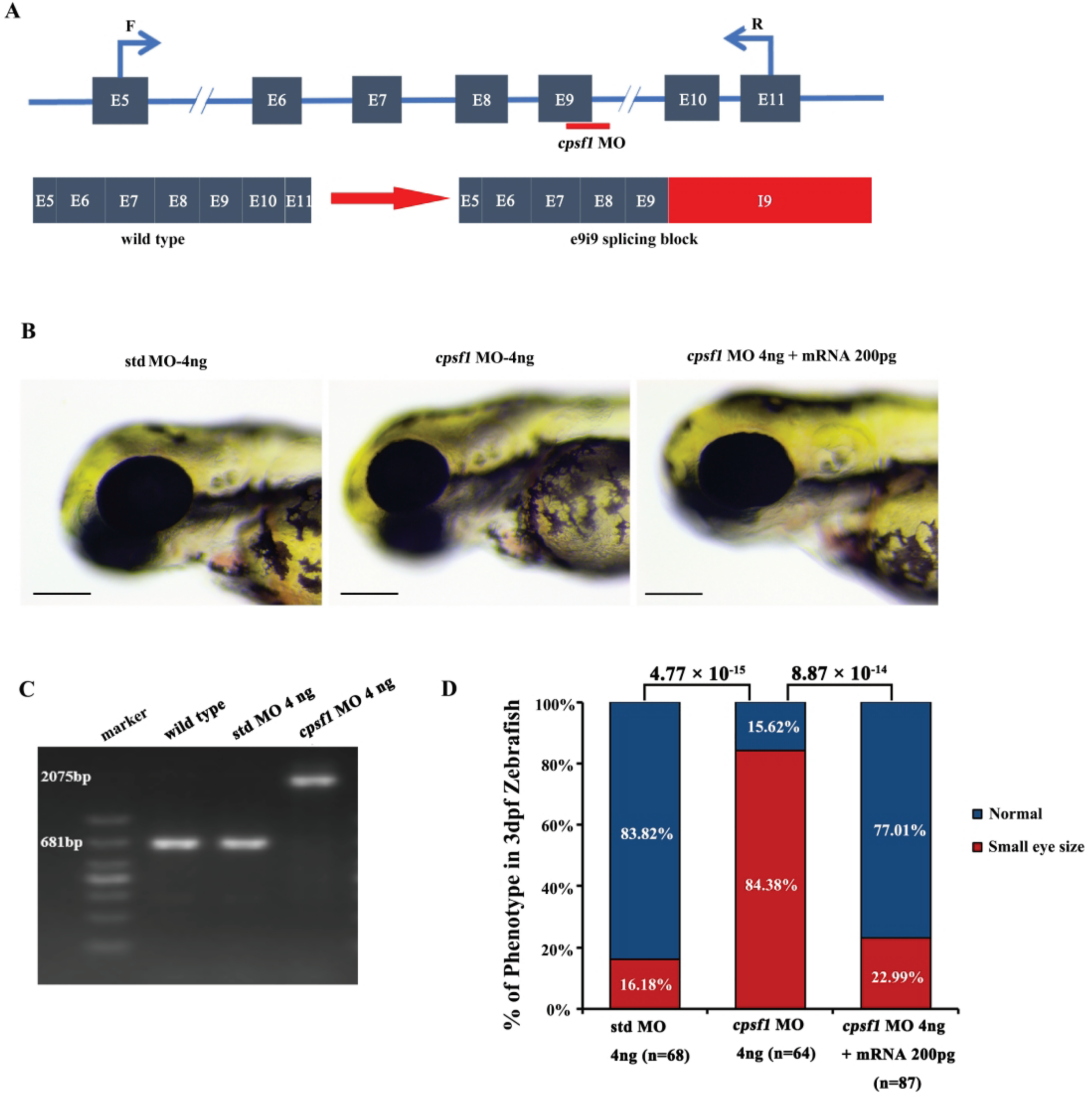
Knockdown of *cpsf1* in zebrafish caused abnormal ocular morphogenesis. **(A)** Diagram illustrating the structure of exons 5 to 11 of the zebrafish *cpsf1* gene, the *cpsf1* MO targeting sites, and the primers used to test splicing products before and after *cpsf1* MO microinjection. **(B)** Eye size in zebrafish at 3 dpf. Left: microinjection of std MO (4 ng), middle: microinjection of *cpsf1* MO (4 ng), right: co-injection of *cpsf1* MO (4 ng) and *cpsf1*-mRNA (200 pg). The eye diameter (eye size) was 294.09 ± 10.28 μm (*n* = 68) for the std control, 263.55 ± 20.85 μm (*n* = 64) for the *cpsf1* morphants and 289.97 ± 15.47 μm (*n* = 87) for the zebrafish subjected to *cpsf1*-MO/*cpsf1*-mRNA co-injection. Small eye size was detected in *cpsf1* morphants, but this phenotype could be rescued by 200 pg of *cpsf1*-mRNA. Std MO-injected zebrafish were used as a control in this study. **(C)** The efficiency of *cpsf1* MO knockdown was tested by RT-PCR using the primers shown in (A). The amplicons from WT and std MO-injected zebrafish RNA were 681 bp, while *cpsf1* MO disrupted the splicing of e9i9, resulting in the occurrence of a larger amplicon of 2075 bp at 24 hours post fertilization (hpf). **(D)** Quantification of small eye size proportions in zebrafish at 3 dpf. The data showed that the proportion of small eye size was significantly increased in *cpsf1* morphants compared to that of the std control (*P* = 4.77 × 10^−15^), and the small eye size phenotype could be rescued by *cpsf1*-mRNA (*P* = 8.87 × 10^−14^) based on the Chi-square test (*P* < 0.017, or 0.05/3, was considered as statistically significant)*.* The number of zebrafish injected in each group is indicated under each column.

**Figure 5:**
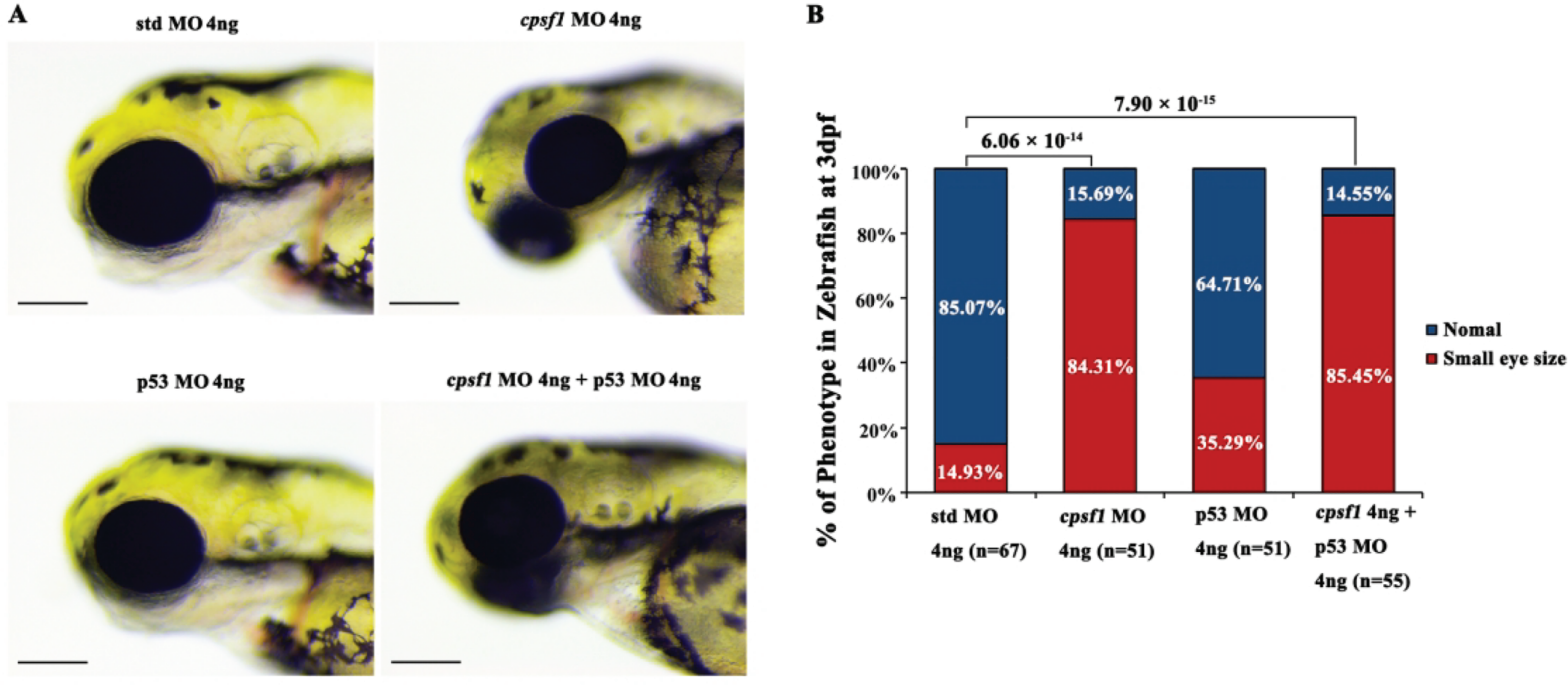
Co-injection of *cpsf1* MO and p53 MO in zebrafish failed to rescue the small eye size phenotype. **(A)** Eye size in zebrafish at 3 dpf. The eye diameter (eye size) was 293.83 ± 10.17 μm (*n* = 67) for the std control, 261.75 ± 21.04 μm (*n* = 51) for the *cpsf1* morphants, and 261.86 ± 19.67 μm (*n* = 55) for the zebrafish subjected to *cpsf1*-MO/p53 MO co-injection. Small eye size was detected in *cpsf1* morphants, but this phenotype could not be rescued by p53 MO. Std MO-injected zebrafish were used as a control in this study. **(B)** Quantification of small eye size proportions in zebrafish at 3 dpf. The proportion of small eye size was significantly increased in *cpsf1* morphants compared to that of the std control (*P* = 6.06 × 10^−14^), but it was still increased compared to that in the std control after co-injection of *cpsf1* MO and p53 MO (*P* = 7.90 × 10^−15^) based on the Chi-square test (*P* < 0.017, or 0.05/3, was considered as statistically significant). The number of zebrafish injected in each group is indicated under each column.

**Figure 6:**
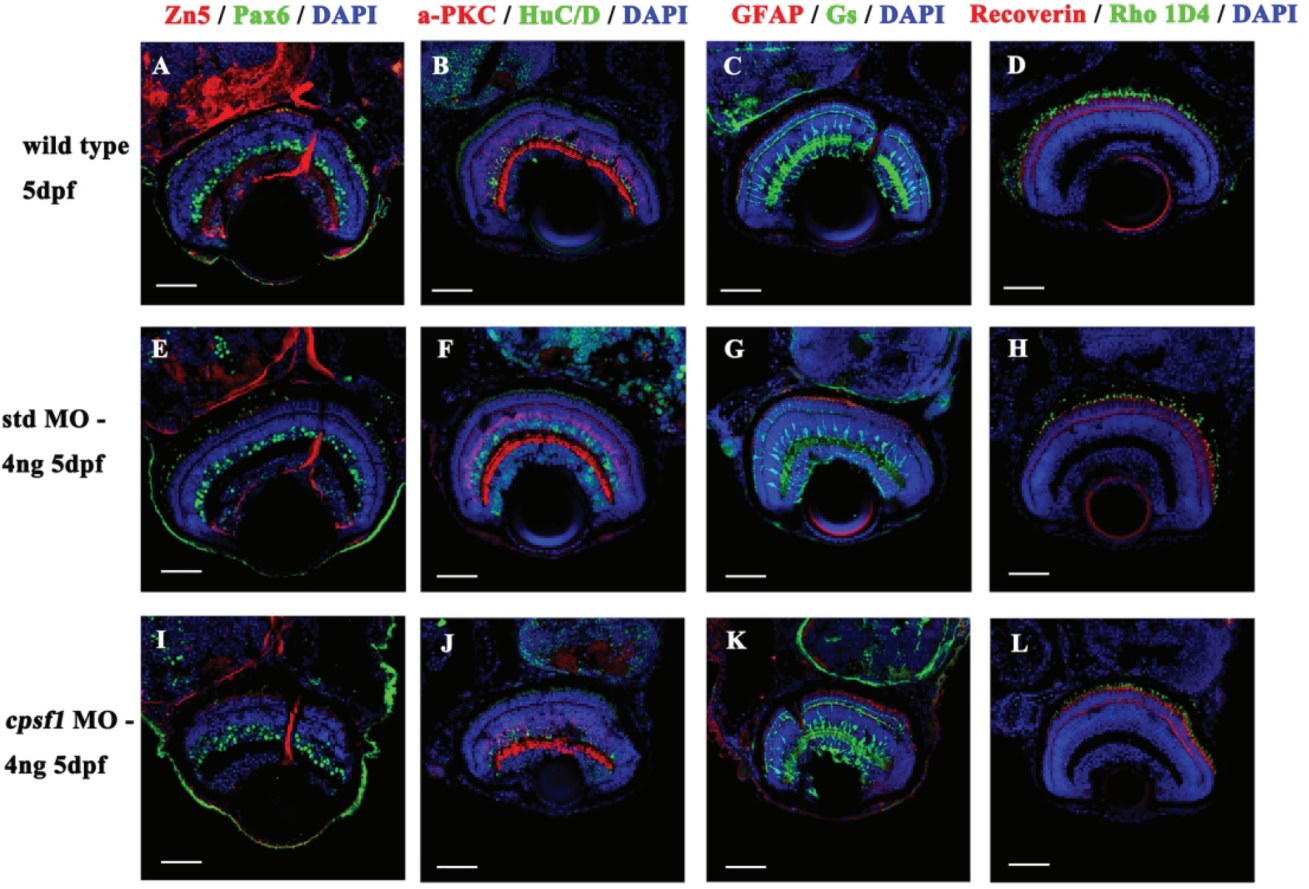
Retinal cells developed normally in WT larvae **(A-D)**, std MO larvae **(E-H)** and *cpsf1* morphants (I–L). (A, E, I) Co-labelling with anti-Pax6 (for ganglion and amacrine precursor cells, green) and anti-Zn5 (for mature RGCs and their axons, red) antibodies showed that the RGCs were mature. (B, F, J) Co-labelling with anti-HuC/D (for ganglion cells and amacrine cells, green) and anti-α PKC (for bipolar cells, red) antibodies indicated that the amacrine cells and bipolar cells developed normally. (C, G, K) Co-labelling with anti-GS (for Müller cells, green) and anti-GFAP (for glial cells and their axons, red) antibodies demonstrated that the Müller cells and glial cells had attained maturity. (D, H, L) Co-labelling with anti-Rho 1D4 (for long double-cone outer segments, green) and anti-Recoverin (for cone bipolar cells, red) antibodies demonstrated that the photoreceptors grew normally.

**Figure 7:**
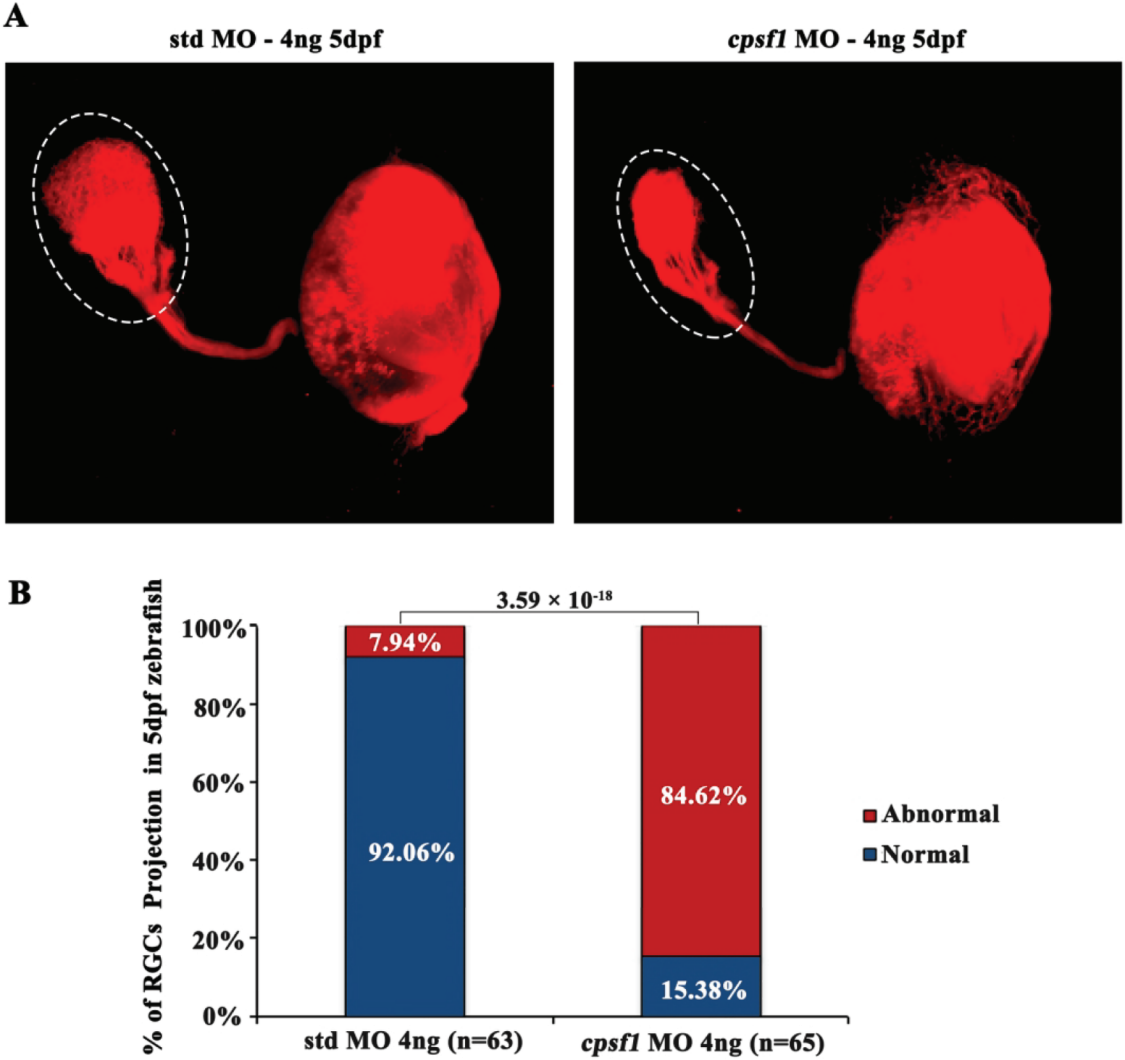
RGC axon projection to the tectum was abnormal in *cpsf1* morphants. **(A)** RGC axon projection in zebrafish at 5 dpf. Left: RGC axon projection in std MO-injected zebrafish, right: RGC axon projection in *cpsf1* morphants. Data showed that *cpsf1* morphants showed less innervation to the tectum than std MO-injected zebrafish. **(B)** Histogram of the percentage of larvae with the phenotype of reduced innervation of the tectum. The area of the tectum with DiI tracing was 18570.60 ± 3969.32 μm^2^ (*n* = 63) in std MO-injected zebrafish, and 8895.07 ± 3756.63 μm^2^ (*n* = 65) in *cpsf1* MO-injected zebrafish. Approximately 84.62% of *cpsf1* morphants displayed reduced area of the tectum, but only 7.94% of std MO-injected zebrafish had this phenotype (*P* = 3.59 × 10^−18^, Chi-square test). The number of zebrafish injected in each group is indicated under each column.

In addition to the six LoF mutations in *CPSF1*, 35 missense variants in *CPSF1* remained in 7 of 623 probands with eoHM and in 28 of 2657 controls after bioinformatics analysis, while 3514 missense variants in this gene remained in gnomAD based on ~230612 alleles after the same analysis. The allele frequencies showed no significant differences among the three groups (*P* = 0.10). However, the frequency of LoF variants was 0.48% (6/1246) in the eoHM samples, which was significantly higher than the 0/5314 frequency in the in-house controls (*P* = 4.60 × 10^-5^, Fisher’s exact test) and the 0.15% (356/230612) frequency in the gnomAD samples (*P* = 0.01). Of the 356 LoF alleles in gnomAD, two variants contributed 110 or 114 alleles. These two variants were absent in East Asian and one of the two is absent in ExAC database. These results suggested that heterozygous LoF mutations in *CPSF1* were specifically and significantly enriched in probands with eoHM. In addition, no LoF alleles were identified in the 2657 controls, whereas four LoF alleles would be expected by chance according to the frequency in gnomAD. This observation could be explained by the fact that the gnomAD database contains individuals with high myopia, whereas the control group in this study did not contain individuals with high myopia**.**

The available clinical data obtained for the eight individuals with LoF mutations were summarized in Table 2. In the six probands, myopia was noticed before school age, and ranged from −6.00 D to −14.00 D. The mother in pedigree HM949 suffered from moderate myopia [−3.50 D for OD (right eye) and − 4.00 D for OS (left eye)], and the age of onset was unavailable (Table 2). Axial length was recorded for six eyes of three probands and ranged from 26.32 mm to 27.09 mm. Fundus changes were typical for high myopia, including optic nerve head crescent and tigroid appearance of the posterior retina (Supplementary Material, Fig. S1A). Electroretinogram (ERG) recordings for the proband of family HM693 showed severely reduced cone responses and mildly reduced rod responses (Supplementary Material, Fig. S1B). No other ocular or syndromic anomalies were mentioned in the case records.

**Table 2:** Clinical data of the probands and available family members with *CPSF1* mutations

**Patient ID**	**Nucleotide change**	**Gender**	**Age at onset**	**Age at exam**	**Visual acuity**		**Refraction (SE)**		**Axial length (mm)**	
					**OD**	**OS**	**OD**	**OS**	**OD**	**OS**
HM337	c.1858C>T	M	12	33	0.02	1.00	−12.00	−14.00	NA	NA
HM635	c.3862_3871dup	F	NA	21	1.20	1.50	−6.50	−7.00	NA	NA
HM653	c.2823_2824del	F	NA	15	1.00	1.00	−10.25	−9.25	NA	NA
HM693	c.15C>G	M	EC	4	0.20	0.30	−9.50	−9.25	26.97	26.74
HM943	c.3823G>T	M	4	7	0.70	0.70	−10.25	−10.50	27.09	27.00
HM943M	c.3823G>T	F	EC	28	0.06	0.10	RD^#^	RD^#^	NA	NA
HM949	c.4146-2A>G	M	3	3	NA	NA	−12.75	−13.50	26.34	26.32
HM949M	c.4146-2A>G	F	NA	29	1.50	1.50	−3.50	−4.00	24.80	25.01

Notes: SE, spherical equivalent; OD, right eye; OS, left eye; F, female; M, male; NA, not available; EC, early childhood.; RD#, she had early onset high myopia but recently had complicated cataract due to uveitis and retinal detachment in both eyes.

### Expression of *CPSF1* in different human tissues

To confirm the expression pattern of *CPSF1* in humans, we performed quantitative reverse transcription polymerase chain reaction (RT-qPCR) to analyse *CPSF1* expression in different human tissues. The results showed that *CPSF1* was highly expressed in human eye structures, including the retina, ciliary body, choroid, optic nerve, sclera and lens (Fig. 3 and Supplementary Material, Fig. S3), indicating that *CPSF1* may function in human eyes, especially in the retina.

### Abnormal eye morphogenesis in *cpsf1* knockdown zebrafish

To determine the potential functional effects of *CPSF1* on the eye, zebrafish were used as a model system. We designed one morpholino oligonucleotide (MO) against the exon 9-intron 9 boundary to interfere with splicing of the *cpsf1* gene, resulting in a truncation of *cpsf1* (Fig. 4A). The intended alteration and its efficiency were verified by reverse transcription-polymerase chain reaction (RT-PCR) using primers designed to flank exon 9 (Supplementary Material, Table S1C). *cpsf1* MO injection resulted in an intron 9 insertion, indicating that the *cpsf1* MO indeed disrupted splicing between exon 9 and intron 9 (Fig. 4C). We then injected *cpsf1* MO and standard control MO (std MO, as a control) at several different doses (2 ng, 4 ng and 6 ng). At 3 days post fertilization (dpf), the zebrafish that were injected with 2 ng of the *cpsf1* MO were normal and similar to the std MO-treated zebrafish (Supplementary Material, Fig. S4). However, in embryos injected with 4 ng of the *cpsf1* MO, the zebrafish eyes appeared small in size at 3 dpf (Supplementary Material, Fig. S4). Moreover, additional severe anomalies that were likely caused by the toxic effects of overdose were produced by injection of 6 ng of *cpsf1* MO; these effects included cardiac oedema, a severely curved tail and body and abnormal pigmentation (Supplementary Material, Fig. S4). Based on these results, the injection of 4 ng of *cpsf1* MO and 4 ng of std MO was selected to study the function of *cpsf1* in zebrafish.

To further demonstrate the specificity of the *cpsf1* MO for *cpsf1*, we co-injected the *cpsf1* MO and the *cpsf1*-mRNA and then scored the eye sizes at 3 dpf. Co-injection of the *cpsf1* MO and the *cpsf1* mRNA significantly reduced the incidence of small eye size from 84.38% to 22.99%, suggesting that this phenotype resulted from the specific knockdown of *cpsf1* (Fig. 4B and D). Since non-specific cell death may also result in small eye size, we co-injected a p53 MO to prevent non-specific cell death (42). The incidence of small eye size was similar between *cpsf1/*p53 MO co-injected zebrafish and the *cpsf1* morphants injected without the p53 MO, suggesting that the phenotype observed in *cpsf1* morphants was not caused by non-specific cell death (Fig. 5). Taken together, these results strongly support the notion that mutation of *cpsf1* leads to abnormal ocular morphogenesis in zebrafish.

Since small eye size is a common phenotype accompanied by retinal degeneration in zebrafish (43–45), we investigated whether *cpsf1*-deficient embryos displayed altered retinal cell development. At 5 dpf, sections of WT or std MO-injected larvae and *cpsf1* morphants were immunolabelled with several antibodies, including an anti-Zn5 antibody for mature RGCs and their axons, an anti-Pax6 antibody for ganglion cells and amacrine precursor cells, an anti-HuC/D antibody for ganglion cells and amacrine cells, an anti-α PKC antibody for bipolar cells, an anti- glutamine synthetase (GS) antibody for Müller cells, an anti- glial fibrillary acidic protein (GFAP) antibody for glial cells and their axons, an anti-Recoverin antibody for cone bipolar cells, an anti-Rho 1D4 antibody for long double-cone outer segments, an anti-Zpr1 antibody for double-cone photoreceptor cells, an anti-Opsin red/green antibody for red/green cone photoreceptor cells, an anti-Opsin green antibody for green cone photoreceptor cells and an anti-Opsin blue antibody for blue cone photoreceptor cells, to evaluate which major retinal cell classes are affected in *cpsf1* morphants. We found that all of the retinal cell classes were present in *cpsf1* morphants. In addition, there were no differences between *cpsf1* morphants and WT larvae and std MO injected larvae, suggesting that mutation of *cpsf1* did not impact mature development in any retinal cell type (Fig. 6 and Supplementary Material, Fig. S5).

### Abnormal axon projection in *cpsf1* knockdown zebrafish

To further investigate whether *cpsf1* contributes to RGC axon projection in zebrafish, we injected DiI (Invitrogen, D282) to trace the RGC axons that project to the tectum at 5 dpf since this axon tract is normally stable at this time. We observed that the tectum of std MO-injected zebrafish was filled with RGC axons and that the optic tract had branched into typical fascicles (Fig. 7A). In *cpsf1* morphants with a mild phenotype, the RGC axon projections were normal (*n* = 10, 10/65) and similar to those observed in WT and std MO-injected zebrafish (data not shown). However, 84.62% of *cpsf1* morphants had a reduced number of projections to the tectum, indicating a role for *cpsf1* in RGC axonal growth in zebrafish (Fig. 7B).

## Discussion

In this study, six heterozygous LoF mutations in *CPSF1* were identified in six of 623 probands with eoHM, and none of these mutations were detected in 2657 probands with other eye diseases; this difference was significant (*P* = 4.60 × 10^-5^, Fisher’s exact test). This difference still existed when the LoF mutations identified in eoHM were compared to those already present in the gnomAD database (*P* = 0.01).

*CPSF1* expression in the human retina and the results obtained after *cpsf1* knockdown in zebrafish further suggest that *CPSF1* plays an important role in retinal function and eye development, which is closely related to eoHM. RT-qPCR revealed relatively high transcription of *CPSF1* in the human retina. The relatively low transcription of *CPSF1* in the human lens might be consistent with axial elongation of the eye rather than curvature myopia in the patients with eoHM in this study. Knockdown of *cpsf1* in zebrafish results in small eye size, and this phenotype could be rescued by *cpsf1*-mRNA, suggesting that *cpsf1* is involved in eye development. Furthermore, the effect of *cpsf1* on eye development might be restricted to the size of the eye, and it may not affect retinal cell differentiation and development because none of the major retinal cell types were affected in *cpsf1* knockdown zebrafish.

The reduced RGC axon projection to the tectum in *cpsf1* knockdown zebrafish is a novel finding, which might be correlated with the retinotectal pathway in refraction. The retinotectal pathway has been shown to play a role in the processes underlying developmental emmetropization (46), and factors that affect emmetropization, including myopia, may change eye size (47) or refraction (48**,**^49^). Reduced thickness has been observed for RGCs and the retinal nerve fibre layer in patients with high myopia (50,51); such reductions may also correlate with reduced RGC projection. In previous studies, retinotectal projection of goldfish was different if the electrophysiological retinotectal map was recorded in water as compared with that in air, where an eye-in-air was expected to have extreme myopia that may affect the projection map (52**,**^53^). Reduced RGC axon projection to the tectum in *cpsf1* knockdown zebrafish might be linked to LoF mutations of *CPSF1* in eoHM, although the exact molecular mechanism has yet to be determined.

A limitation of the current study is that the small eye phenotype in zebrafish is inconsistent with axial elongation of the eyes in humans. At present, we do not know exactly why different phenotypes are observed between zebrafish and humans. Similarly, mutations in *RP2* cause retinitis pigmentosa accompanied by eoHM in humans, but knockdown of *rp2* in zebrafish resulted in small eye size (54). Furthermore, different mutations in the same gene cause contrasting phenotypes. For example, mutations in *PAX6* lead to microphthalmia in most patients but can also cause high myopia associated with axial elongation (55).

Of the six probands with *CPSF1* mutations in this study, five did not have mutations in other genes known to be associated with genetic eye diseases, but one (HM693) had a c.6980C>T (p.S2327F) variant in *PRPF8*, as described in our previous study (34). Mutations in *PRPF8* have been associated with autosomal dominant retinitis pigmentosa (adRP) with rod-dominated defects (56,57). However, ERG recordings from individual HM693 showed cone-dominated defects (Supplementary Material, Fig. S1) which suggested the c.6980C>T variant in *PRPF8* was not causative. In addition, both truncation and missense mutations in *PRPF8* have been reported to cause adRP, but the pathogenicity of missense mutations in *PRPF8* is suspicious in some cases since missense variants in *PRPF8* are common based on the gnomAD database; rare damaging variants in *PRPF8* had a sum allele frequency of 2.17 × 10^−3^ (614/282896), which is higher than the prevalence of RP (1/3000). Therefore, the LoF variants in *CPSF1* were more likely to be causative than the c.6980C>T variant in *PRPF8* in HM693. In fact, we have mentioned in our previous study that some of the mutations in RetNet genes that were identified in our previous studies may not necessarily be causative (34).

In summary, LoF mutations in *CPSF1* were found in six of 623 unrelated families with eoHM but none of 2657 probands with other forms of genetic eye diseases, indicating an association of LoF of *CPSF1* with eoHM. *CPSF1* was highly expressed in the retina, suggesting an important role in retinal function. Knockdown of *cpsf1* resulted in a change of eye size in zebrafish, implying a role in regulating eye size. Furthermore, a reduced number of retinotectal projections in *cpsf1*-knockdown zebrafish might suggest that the eoHM could be possibly a result from changes of the visual pathway triggered by *cpsf1* defects. These lines of evidences support that *CPSF1* mutations are associated with eoHM and involved in RGC axon projection. Our results may provide new avenue for further exploring the molecular pathogenesis of eoHM, as well as the functions of *CPSF1* in eye development and visual pathway although further studies are expected to confirm current findings.

## Materials and Methods

### Patients and clinical data

Probands from 623 families with eoHM or 2657 families with other forms of genetic eye diseases were collected, and some of these probands were described previously (34,35,58,59). The 2657 probands included 1139 with retinal degeneration, 812 with glaucoma, and 706 with other genetic eye diseases. Written informed consent conforming to the tenets of the Declaration of Helsinki and following the Guidance for Sample Collection for Human Genetic Diseases (863-Plan) of the Ministry of Public Health of China was obtained from all participating individuals or their guardians prior to the collection of clinical data and peripheral venous blood. This study was approved by the Institutional Review Boards of the Zhongshan Ophthalmic Center.

### Searching WES data for a novel candidate gene

WES data from 623 probands with eoHM were previously analysed by our group (34,35). The WES data from these 623 probands were compared to in-house data from 2657 probands with other forms of genetic eye diseases. A few novel candidate genes were identified, including *CPSF1*, in which specific mutations were identified exclusively in probands with eoHM. All variants of *CPSF1* were collected and investigated through multi-step bioinformatics analysis, as described in our previous reports (34).

### Sanger sequencing

Sanger sequencing was used to validate potential pathogenic variants. The primers used to amplify and sequence the fragments harbouring candidate variants were designed in Primer 3 (http://primer3.ut.ee/) (Supplementary Material, Table S1A). The methods used for amplification, sequencing and analysis of the target fragments have been previously described (60).

### Minigene analysis

Two minigenes were constructed *in vitro* covering the two mutation sites, c.4146-2A>G and c.3823G>T, and their 5′ and 3′ adjacent regions (Fig. 2 and Supplementary Material, Fig. S2). The sequences of the WT and mutant minigenes were amplified by PCR from patients with mutations and normal controls using primer containing restriction enzyme sites (Supplementary Material, Table S1C). The amplicons were cloned into the pEGFPN1 vector containing the cytomegalovirus (CMV) promoter and the Enhanced Green Fluorescent Protein (EGFP) protein. The recombined plasmids were transfected into HEK293T cells by calcium phosphate transfection and cultured for 24 h at 37°C. cDNA was reverse transcribed from RNA extracted from the transfected HEK293T cells. The cDNA sequences were examined by Sanger sequencing.

### qPCR

Total RNA samples were prepared from 32 human tissues, including 24 samples purchased from TaKaRa (Japan), and RNA samples from 12 male and female Caucasian individuals who experienced sudden death at the age of 18 to 54 years were pooled. An additional eight samples were obtained from an eye donor who died of meningioma. cDNA was prepared from RNA isolated from the 32 tissues using a PrimeScript™ RT reagent kit (RR047A; TaKaRa; Japan). Quantitative primers (Supplementary Material, Table S1C) were designed in Primer 3 (http://primer3.ut.ee/) as previously described. PowerUp SYBR Green Master Mix (A25742, Applied Biosystems; Thermo Fisher Scientific; Massachusetts) was used for qPCR. The ACTB gene, which encodes beta actin, was selected as a control, and fold changes in RNA levels were calculated by the ΔΔCt method, as previously described (61).

### Knockdown of *CPSF1* in zebrafish

All WT zebrafish (AB *Danio rerio*) embryos were bred through natural spawning, raised and maintained at 28.5°C with a 14 h light:10 h dark cycle and staged in hpf. All zebrafish were obtained from the Zebrafish Model Animal Facility at the Institute of Clinical and Translational Research of Sun Yat-sen University. The developmental stages of the embryos were determined based on the morphological features of the eye.

The following MOs were designed and synthesized by Gene-Tools, LLC (Corvallis, OR, USA): the *cpsf1* MO (targeting splicing at e9i9, as described in a previous study (40), std MO (62) and p53 MO (Supplementary Material, Table S1B). All of the MOs were diluted in distilled water. All data presented in this study were obtained from embryos in which 2 ng, 4 ng or 6 ng of the *cpsf1* MO or std MO was microinjected into the yolk at the 1-cell stage.

To test the efficiency of the *cpsf1* MO, RNA was isolated from WT, std MO and *cpsf1* MO embryos at 24 hpf. cDNA was synthesized from these RNA samples by RT-PCR and amplified by using primers crossing the MO sequence that were designed in Primer 3 (Supplementary Material, Table S1C).

The full-length cDNA of *cpsf1* in zebrafish was amplified by PCR and cloned into the pSC2+ vector. *cpsf1* mRNA was synthesized by using an mMESSAGE mMACHINE™ SP6 kit (Am1340; Invitrogen; California). The mRNA was diluted to 200 pg and co-injected with the *cpsf1* MO (4 ng) into the yolks of embryos at the 1-cell stage.

Equivalent amounts of a p53 MO and *cpsf1* MO (4 ng each) were also co-injected to inhibit non-specific cell death (42).

The eye sizes of embryos were measured by determining the length of the eye from the nasal edge to the temporal edge using ImageJ software. The proportions of larvae with small eye size were compared in different groups by a Chi-square test in SPSS 23.0 software (International Business Machines Corporation, New York; USA). It was considered to be statistically significant if the *P*-value was less than 0.017 (0.05/3) after Bonferroni correction. Small eye size in this study was defined as an eye diameter less than the mean - standard error (SE) of the eye diameter of control larvae, while normal eye size indicates that the eye size of the larvae was within the mean ± SE of that of control larvae. All the fish groups used in this study are based on three independent replicates, and the numbers and statistical analysis (mean, SE) are based on the total data from all three experiments.

Immunofluorescence was performed using frozen sections of embryos obtained at 5 dpf. The sections were subjected to antigen retrieval at 98°C for 30 min except for those analysed with anti-Zn5 antibodies (1:500; Zebrafish International Resource Center [ZIRC]; Oregon). The sections were then incubated with primary antibodies for 16 h at 4°C. The primary antibodies used in this study included anti-Zn5, anti-HuC/D (1:100; A-21271; Invitrogen; California), anti-Pax6 (1:500; PRB-278P-100;Covance; Oregon), anti-αPKC (1:500; sc-208; Santa Cruz Biotechnology; California), anti-GS (1:1000; 610518; BD Biosciences; New Jersey), anti-GFAP (1:1000; PA5-16291; Thermo Fisher Scientific; Massachusetts), anti-Recoverin (1:500; AB5585; Millipore; Massachusetts), anti-Rho 1D4 (1:500; ab5417; Abcam; Cambridge, UK), anti-Zpr1 (1:500; ab174435; Abcam; Cambridge, UK), anti-Opsin red/green (1:500; AB5405; Millipore; Massachusetts), anti-Opsin green (1:500; OSR00222W; Thermo Fisher Scientific; Massachusetts), and anti-Opsin blue (1:500; AB5407; Millipore; Massachusetts) antibodies. The following secondary antibodies were used: Alexa Fluor 488-conjugated goat anti-mouse IgG antibodies (1:500; Cell Signaling Technology 4408) and Alexa Fluor 555-conjugated goat anti-rabbit IgG antibodies (1:500; 4413; Cell Signaling Technology; Massachusetts). Images were captured with a Nikon C2 confocal microscope (Nikon Eclipse Ni-E, Japan).

Embryos were treated with 1× phenylthiourea (PTU, Sigma-Aldrich Corp., St. Louis, MO, USA) starting at 24 hpf to prevent pigmentation and fixed in 4% paraformaldehyde (4% PFA) at 5 dpf. DiI (D282; Invitrogen; California) was diluted in dimethylsulfoxide and injected into the retina. The embryos were then incubated at 4°C for 24 h when the dye had diffused to the tectum. RGC axon projection was captured with the z-axis of an LSM 780 confocal microscope (Zeiss, Germany). The area of tectum was measured by Image J software. The proportions of larvae with abnormal RGC axon projection area were compared in different groups by a Chi-square test in SPSS 23.0 software (International Business Machines Corporation). It was considered to be statistically significant if the *P* value less than 0.05. The mean and SE of projection area in two groups of larvae were obtained, including *cpsf1* MO-4 ng treating larvae and std MO-4 ng treating larvae. It was defined as reduced projection if the projection area was less than the mean - SEs of the mean of that of control larvae.

## Supplementary Material

Supplementary Data
