# Supplementary material for: CPSF1 mutations are associated with early-onset high myopia and involved in retinal ganglion cell axon projection
Source: Hum Mol Genet. 2019 Jan 26;28(12):1959–70. doi: 10.1093/hmg/ddz029 (PMC6548346; doi:10.1093/hmg/ddz029)
Supplement: Supplementary Data [file ddz029_supp.zip › ddz029_Suppl.docx]

**Figure legends for supplementary figures S1-S6**

**Figure S1.** Available fundus changes of the probands HM693 and HM949 and ERG recording of the proband HM693. (A) Typical fundus changes of high myopia, including an optic nerve head crescent and a “tigroid” appearance of the posterior retina. (B) ERG recording of the proband HM693 showed severely reduced cone responses and mildly reduced rod responses.

**Figure S2.** Minigene analysis of the effect of the c.3823G>T mutation for splicing. (A) The c.3823G>T mutation located at E33. The minigene construction including E33 and flanking IVS32 and IVS33 by minigene analysis as well as pEGFPN1 construct. Arrow, the location of the c.3823G>T mutation. (B) Sequences of cDNA by minigene analysis. The mutated allele and wild type allele both resulted in the inclusion of IVS32. (C) Alignment of the amino acid sequences showed highly conservation of the aspartic acid at 1275 among nine vertebrates.

**Figure S3**. Expressionanalysis of *CPSF1* mRNA in human ocular tissues. All of the RNA samples used in this study except for the RPE sample were acquired from an eye donor who died of meningioma at the age of 17 years old. The RPE sample was acquired from the ARPE-19 cell line.

**Figure S4**. Knockdown of *cpsf1* at different doses in zebrafish caused differences in ocular morphogenesis. (A) Phenotype of MO-injected zebrafish. Upper: microinjection with 2 ng, 4 ng, or 6 ng of std MO; lower: microinjection with 2 ng, 4 ng, or 6 ng of *cpsf1* MO. The data showed that body and eye size were normal after injecting 2 ng, 4 ng, or 6 ng of std MO. Three phenotypes were observed in the *cpsf1* morphants and classified based on degree (mild, moderate or severe) following injection with 2 ng, 4 ng or 6 ng of *cpsf1* MO. (B) Quantiﬁcation of small eye size proportions in zebrafish at 3 dpf. The eye diameter (eye size) was 290.58 ± 17.94 μm (n=45) for zebrafish injected with std MO (2 ng) and 289.58 ± 28.84 μm (n= 60) for zebrafish injected with *cpsf1* MO (2 ng); 285.28 ± 12.25μm (n=51) for zebrafish injected with std MO (4 ng) and 250.03 ± 32.31 μm (n=74) for zebrafish injected with *cpsf1*-MO (4 ng); and 285.06 ± 15.80 μm (n=57) for zebrafish injected with std MO (6 ng) and 212.64 ± 28.68 μm (n=83) for zebrafish injected with *cpsf1*-MO (6 ng). The data showed that the proportion of small eye size was significantly increased in *cpsf1* morphants injected with 4 ng and 6 ng of *cpsf1* MO than in those injected with the std control (*P*= 4.66 × 10^-10^ for 4 ng and *P*= 9.17 × 10^-17^ for 6 ng), but there was no difference between the *cpsf1* morphants injected with 2 ng of *cpsf1* MO and those injected with the std control (*P* = 0.88) based on Chi-square test (*P*<0.05 was considered as statistically significant). The number of zebraﬁsh injected in each group is indicated under each column.

**Figure S5**. Photoreceptor cells developed normally in wild-type larvae (A-C), std MO larvae (D-F), and *cpsf1* morphants (G-I). (A, D, G): Co-labelling with anti-Zpr1 (for double-cone photoreceptor cells, green) and anti-Opsin Blue (for blue cone photoreceptor cells, red) antibodies showed that the blue cone photoreceptor cells were mature. (B, E, H): Co-labelling with anti-Zpr1 (for double-cone photoreceptor cells, green) and anti-Opsin Red/Green (for red/green cone photoreceptor cells, red) antibodies indicated that the red and green cone photoreceptor cells developed normally. (C, F, I): Co-labelling with anti-Zpr1 (for double-cone photoreceptor cells, green) and anti-Opsin Green (for green cone photoreceptor cells, red) antibodies demonstrated that the green cone photoreceptor cells attained maturity.

**Figure S6.** The 76bp sequence of whole IVS35 in the c.4146-2A>G mutated allele.
