# Supplementary figures and images for: CPSF1 mutations are associated with early-onset high myopia and involved in retinal ganglion cell axon projection
Source: Hum Mol Genet. 2019 Jan 26;28(12):1959–70. doi: 10.1093/hmg/ddz029 (PMC6548346; doi:10.1093/hmg/ddz029)

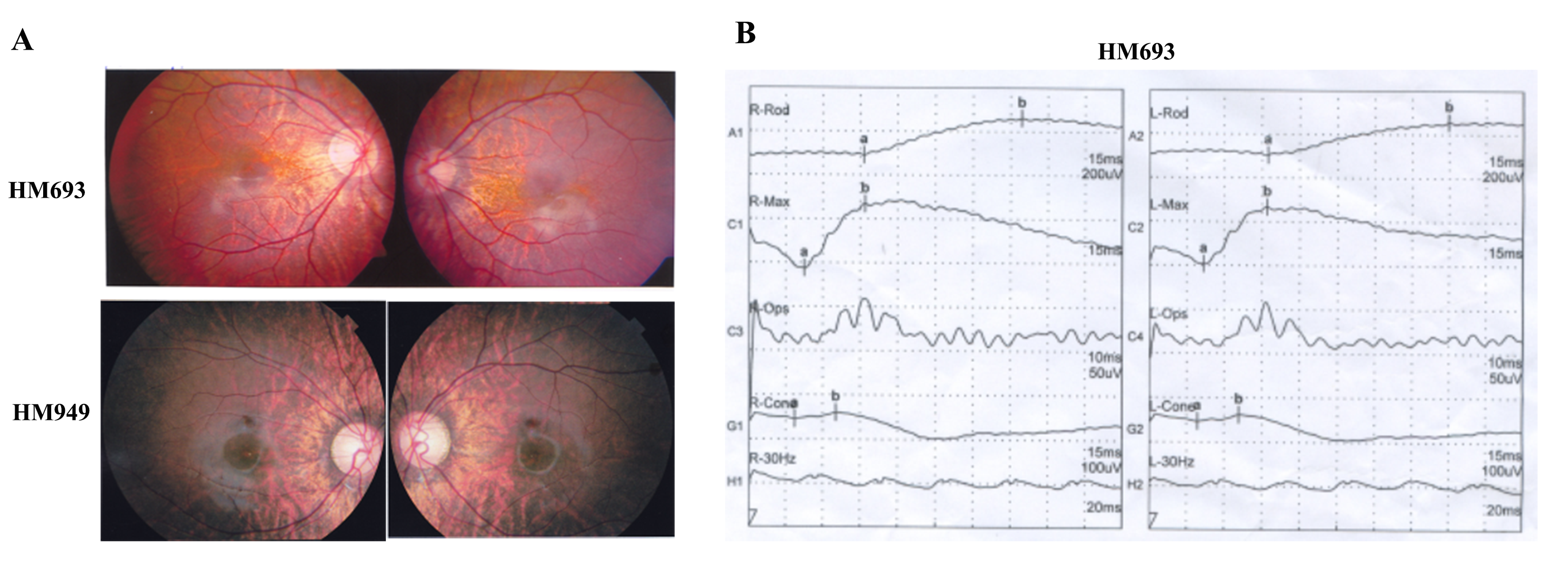

Supplement: Supplementary Data [file ddz029_supp.zip › OuyangJ-Figure S1-Fundus.tif]

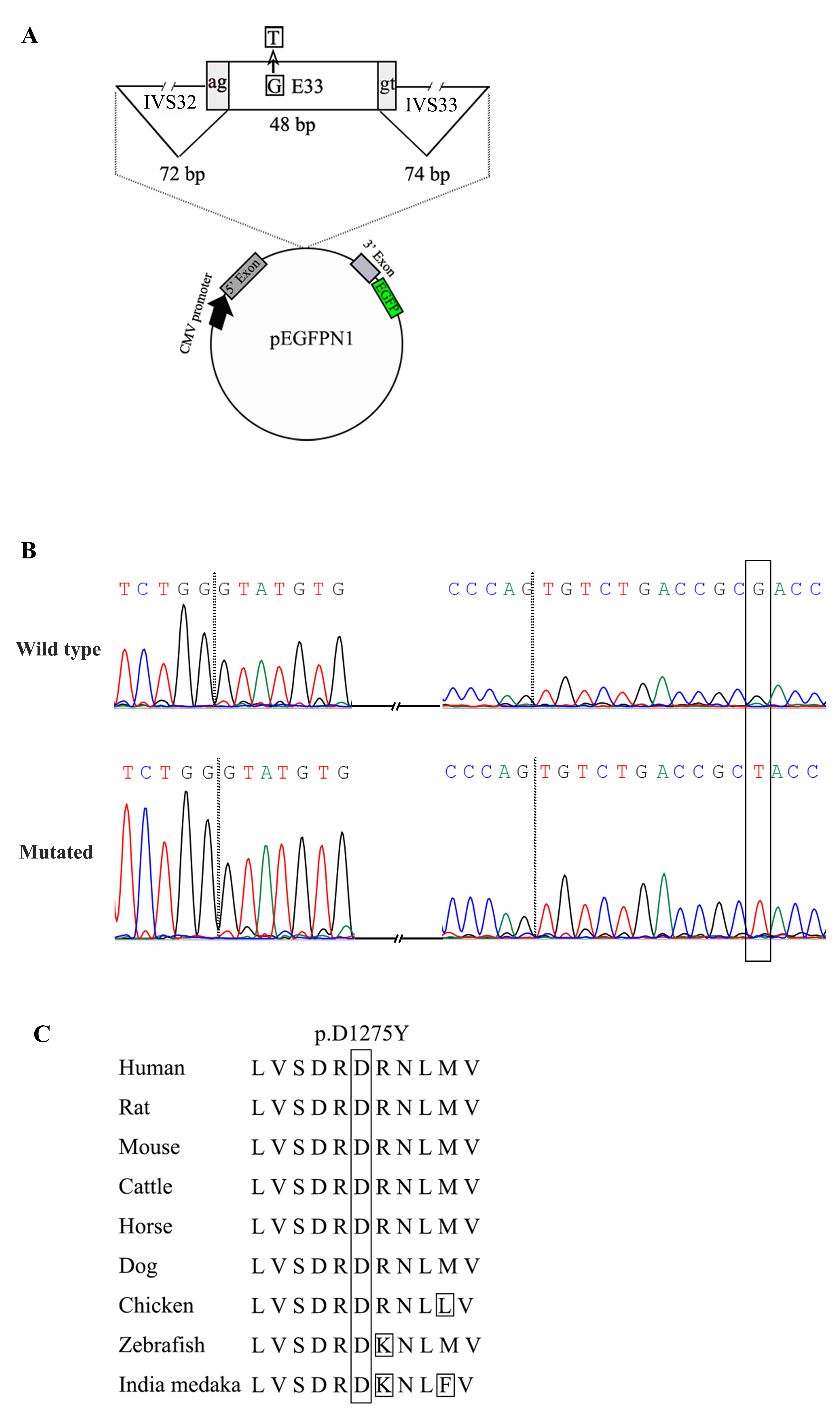

Supplement: Supplementary Data [file ddz029_supp.zip › OuyangJ-Figure S2-Minigene of HM943.tif]

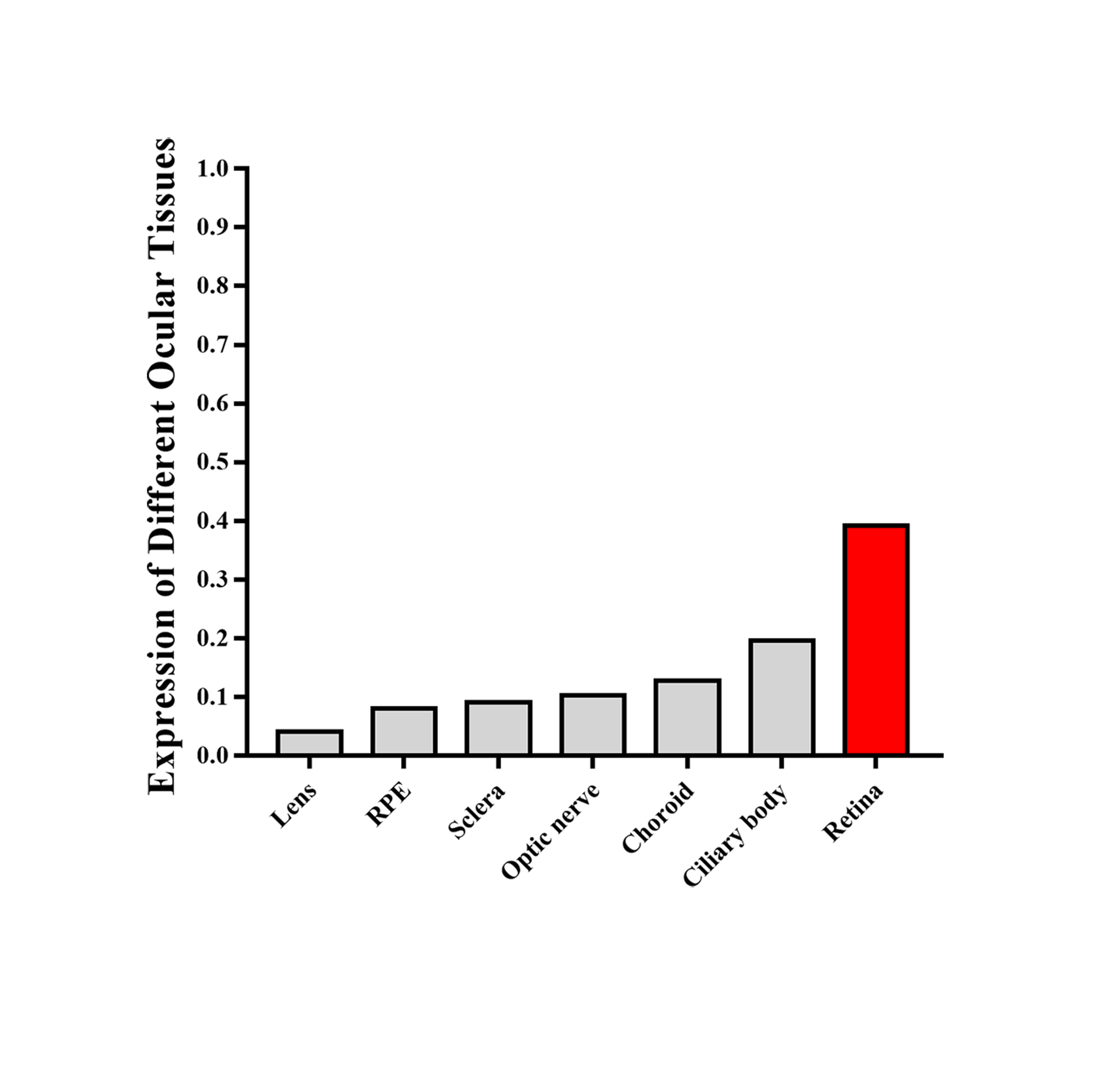

Supplement: Supplementary Data [file ddz029_supp.zip › OuyangJ-Figure S3-Expression in human ocular.tif]

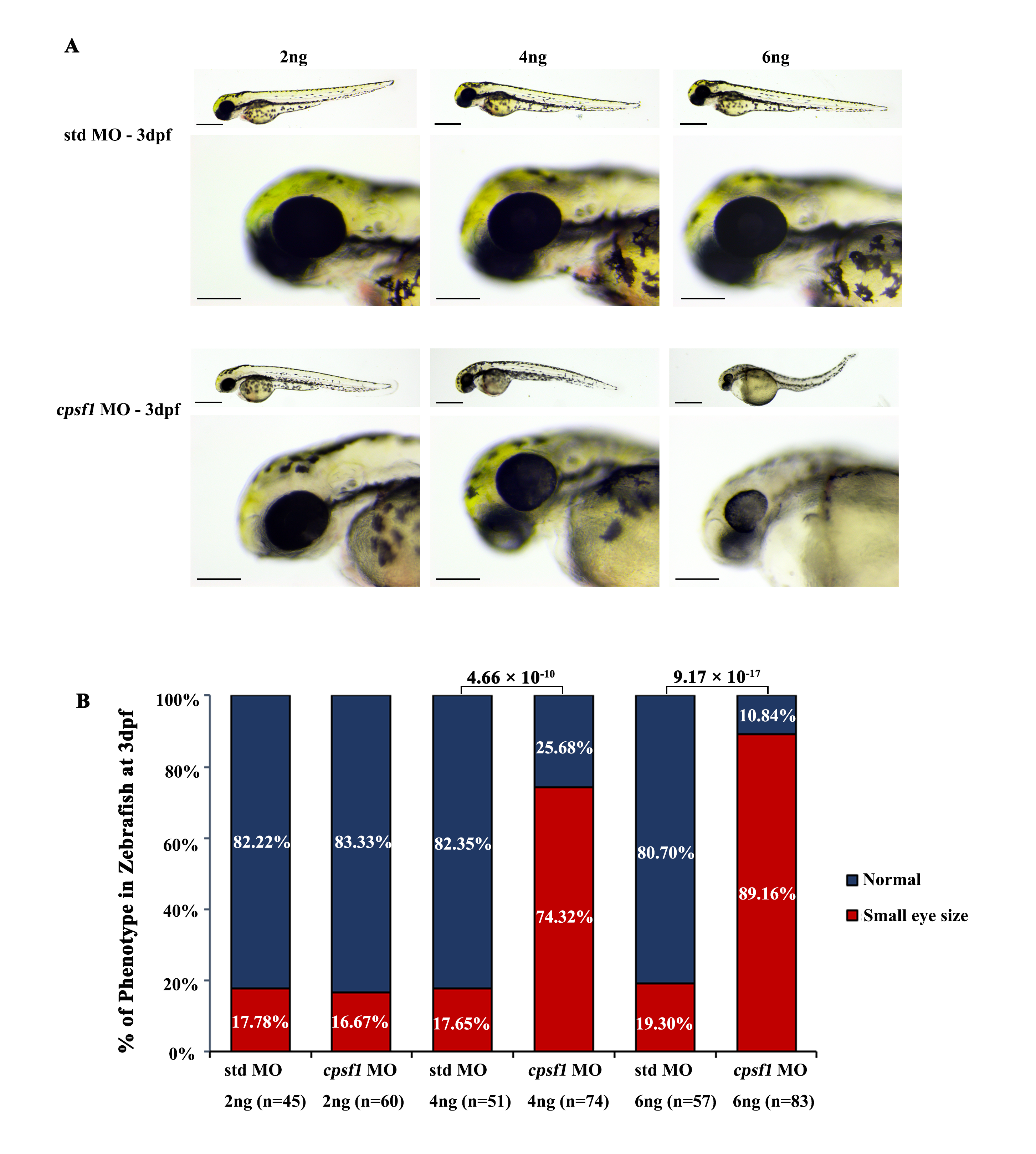

Supplement: Supplementary Data [file ddz029_supp.zip › OuyangJ-Figure S4-Different MO doses-final.tif]

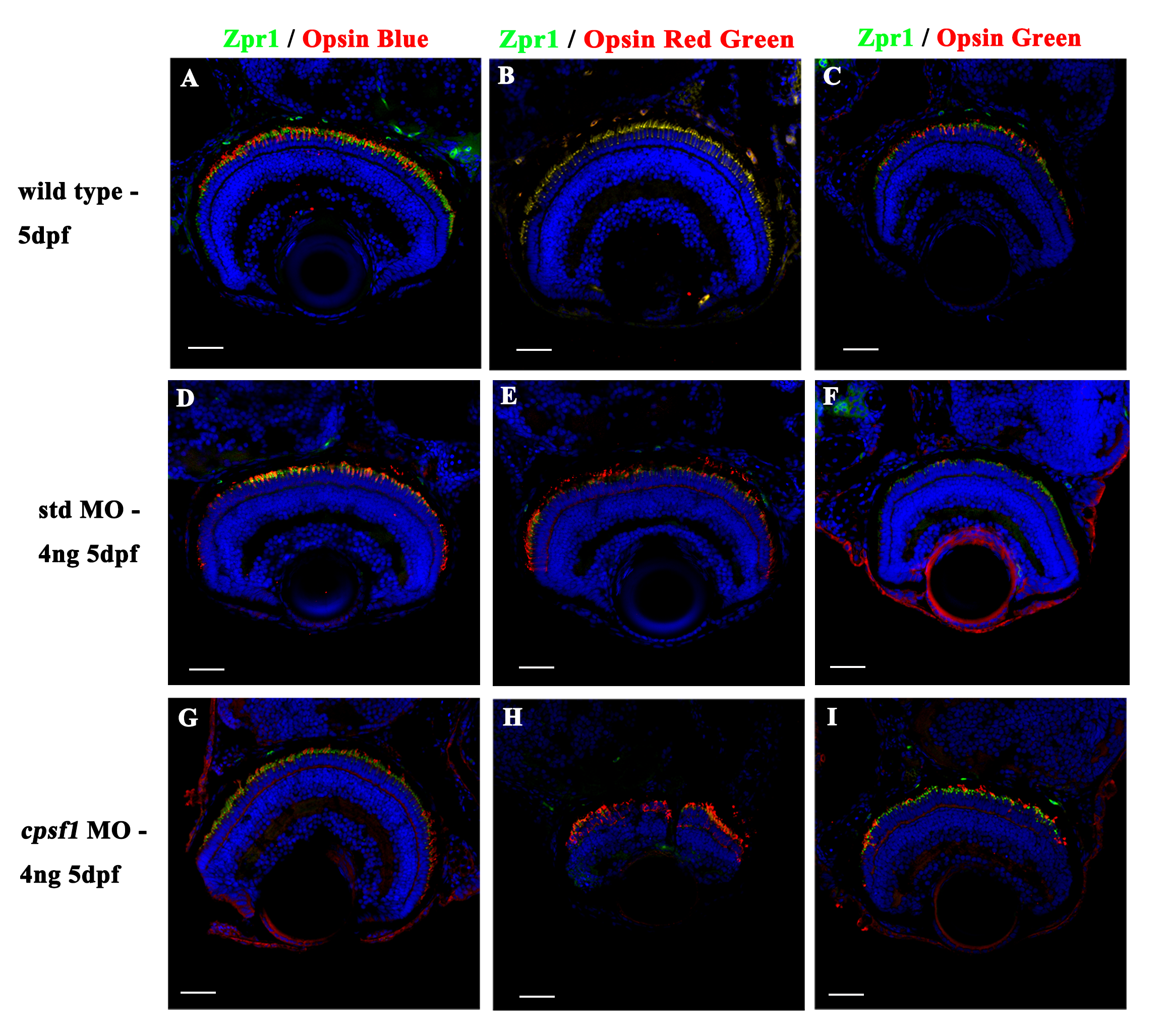

Supplement: Supplementary Data [file ddz029_supp.zip › OuyangJ-Figure S5-Photoreceptors in cpsf1 morphant-final.tif]

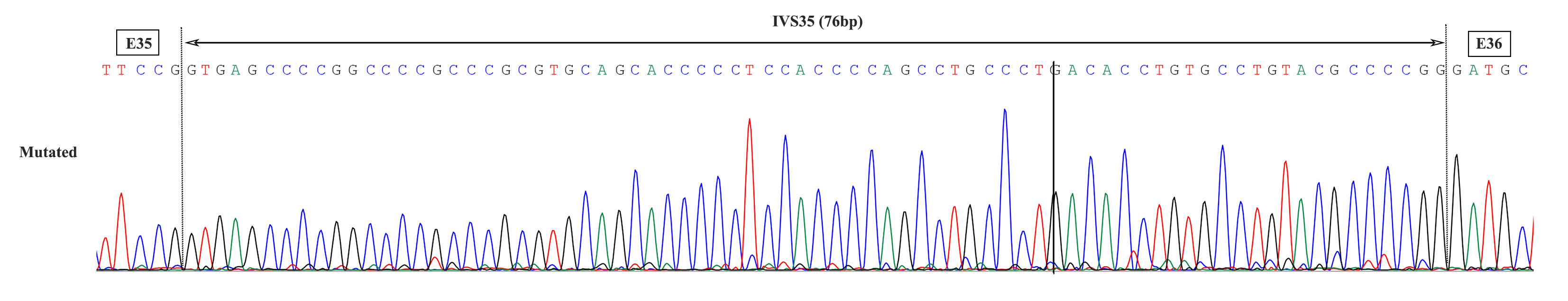

Supplement: Supplementary Data [file ddz029_supp.zip › OuyangJ-Figure S6. IVS35 (76bp) of HM949.tif]
